# Tumor Cell Plasticity and Stromal Microenvironment Distinguish Papillary and Follicular Growth Patterns in a Mouse Model of BRAFV600E-Induced Thyroid Cancer

**DOI:** 10.1158/2767-9764.CRC-24-0474

**Published:** 2025-03-07

**Authors:** Elin Schoultz, Carmen Moccia, Shawn Liang, Ellen Johansson, Mikael Nilsson

**Affiliations:** 1 Sahlgrenska Center for Cancer Research, Institute of Biomedicine, University of Gothenburg, Göteborg, Sweden.; 2 Department of Medical Chemistry and Cell Biology, Institute of Biomedicine, University of Gothenburg, Göteborg, Sweden.

## Abstract

Differentiated thyroid cancer primarily classified by tumor histology comprises follicular thyroid carcinoma (FTC) and papillary thyroid carcinoma (PTC), which represent distinct malignancies regarding the pattern of tumor spreading, responsiveness to radioiodine treatment, and clinical outcome. As FTC and PTC also differ genetically i.e., *RAS* mutations predominate in FTC whereas mutant *BRAF* is much more frequent in PTC, it is assumed although yet unproven that the archetypical tumor growth pattern – follicular versus papillary – depends on mutation identity and potentially the graded signaling output of the MAPK pathway that differs in response to constitutive activation by RAS or BRAF. In this study, we show by clonal tracing in mice carrying a *Braf* mutant allele with targeted expression of BRAFV600E exclusively in the thyroid gland that sporadically developed tumors either adopt a follicular or a papillary phenotype depending on follicle origin. Moreover, a single tumor clone may transit from papillary to follicular growth associated with asymmetric distribution of stromal tissue in the immediate vicinity of the tumor. Key to these observations is monitoring the progeny of cells undergoing spontaneous recombination of both *Braf*^*CA*^ and the reporter gene because of Cre leakage which, in contrast to after induced oncogene activation comprising all target cells, implies that tumors develop stochastically and asynchronously originating from a limited number of BRAF mutant cells and taking place within a preserved thyroid tissue microenvironment. The results suggest that the natural heterogeneity of follicles and tumor cell plasticity modify BRAFV600E-induced neoplastic growth leading to divergent tumor histogenesis.

**Significance:**

Cell-of-origin intrinsic features rather than driver mutation identity influence tumor growth patterning in differentiated thyroid cancer and might impact histopathologic diagnosis of thyroid carcinoma subtypes.

## Introduction

Differentiated thyroid cancer (DTC) mainly comprises two histologically distinct tumor entities, papillary thyroid carcinoma (PTC) and follicular thyroid carcinoma (FTC), which display different clinical features and for most cases are associated with distinct pathogenic mutation profiles (1). Both tumor types can progress to anaplastic thyroid cancer, although the originating neoplastic lesion is always characterized by genomic simplicity (2). PTC is the most common thyroid malignancy, and *BRAF* mutation encoding BRAF^V600E^ oncoprotein is the primary driver mutation in majority of tumors (3). FTC, occurring less frequently, is predominantly associated with *RAS* mutations (4, 5). Routine pathology diagnosis is still primarily morphologic and a challenge also for the trained eye, e.g., to differentiate between neoplastic benign and cancerous lesions presenting with a follicular phenotype (5). The principal differences in tumor growth pattern – papillary versus follicular – is intriguing and cannot easily be attributed to the differential pathogenicity of mutant RAS and BRAF of which both confer constitutive activation of the MAPK signaling pathway. Hence, the follicular variant of PTC is encountered among both BRAF and RAS mutant thyroid cancers (6, 7), and BRAF mutation is occasionally detected as the single oncogenic event in FTC (8, 9).

In a new mouse model of sporadic PTC generated by stochastic activation of a *Braf* mutant allele, we recently found that multifocal tumors of different clonality possess highly variable morphologies and growth rates reproducing at least partially the spectrum of PTC subtypes being diagnosed in humans with DTC (10). The results suggest that thyroid cells respond differently to BRAF activation and infer that follicular heterogeneity, a well-known feature of the thyroid gland both functionally and histologically (11–15), might influence tumor heterogeneity. In this study, by concomitant activation of *Braf*^*CA*^ and a double-fluorescent reporter, we were able to trace the fate of BRAF mutant clones and capture and characterize morphogenetic traits that govern the generation of papillary and follicular tumor phenotypes *in vivo* elicited by one and the same driver mutation.

## Materials and Methods

### Mouse strains and lineage tracing experiments

*Tg-CreER* ^*T2*^ mice (RRID: IMSR_JAX:030676) with inducible CRE recombinase conditionally expressed under control of the thyroglobulin (*Tg*) promoter (16) were double-crossed with *Braf*^*CA*^ heterozygous mice (RRID: IMSR_JAX:017837; ref. 17) and *mTmG* double-fluorescent reporter mice (RRID: IMSR_JAX:037456; ref. 18). Strains were backcrossed with C57BL/6J mice (obtained from Charles River Laboratories) at least 10 generations before recombination. Ear skin biopsies were sampled for genotyping with PCR along with ID-labeling of mice at 3 to 4 weeks of age. Heterozygous *Tg-CreER*^*T2*^;*Braf*^*CA/+*^;*mTmG* mice were subjected to lineage tracing of clonal tumor growth after spontaneous Cre-mediated coactivation of transgenes in the thyroid gland as monitored by fluorescence microscopy at 3, 6, and 12 months of age. In control experiments of global transgene activation, tamoxifen dissolved in sunflower oil (10 mg/mL) was injected intraperitoneally (50 μL) daily ×4, after which thyroid glands were excised and examined after 10 days or 3 months for induced oncogene and reporter gene coactivation. Animal experiments were approved by the regional Ethics Committee (Dnr 5.8.18-03925/2018 and 5.8.18-04502/2023) according to European standards and national regulations provided by the Swedish Agriculture Agency. Both male and female mice were included in experiments. Animals were allocated to groups (time endpoints and induced vs. noninduced conditions) from multiple breeding and litters. Possible variations due to sex were not investigated.

### Histology, IHC, and fluorescence microscopy

After sacrifice, thyroids excised *en bloc* with the trachea, esophagus, and infrahyoid muscles were fixed in 4% paraformaldehyde and processed for paraffin sections and routine hematoxylin–eosin staining. Deparaffinized sections were subjected to epitope retrieval by PT Link (Agilent Technologies) and quenching of endogenous peroxidase activity prior to immunostaining optimized with the Dako EnVision system for antibodies (source: titer) against *Tg* (Agilent, Cat. # A 0251, RRID: AB_10013723; 1:5,000) and CRE recombinase (Cell Signaling Technology, Cat. # 15036, RRID: AB_2798694; 1:125). Sections were viewed and imaged in an Olympus BX45TF microscope equipped with a Nikon DS-U2 camera. Fixed tissue samples were incubated in 30% sucrose overnight, embedded in optimal cutting temperature (OCT) compound Tissue-Tek (Sakura Finetek), and saved at −80°C. Cryosections were collected on SuperFrost glass slides (Vector) and counter-stained with 4′,6-diamidino-2-phenylindole (DAPI) nuclear stain (Sigma-Aldrich) before mounting with fluorescence mounting medium (Agilent). Fluorescence (mTomato and eGFP) was analyzed using a Zeiss Axioskop 2 plus microscope equipped with a Nikon DS-Qi1Mc camera. Image acquisition and processing used the NIS-Elements Imaging Software.

### Morphometry and animation

The percentage of mG^+^ thyroid cells was quantified on cryosections of thyroids (*n* = 3 per group) from 6-month-old *Tg-CreER*^*T2*^;*Braf*^*CA/+*^;*mTmG* mice with or without subacute tamoxifen injections as indicated. Specimens were transversely serial-sectioned encompassing, in different experiments, the entire gland or at three standardized levels: equator and halfway to either lobe pole. Cell counting followed a predetermined field of view–based protocol for microscopic evaluation at ×20 magnification. Microscopists were blinded to genotype and treatments of specimens subjected to morphometric analysis. 3D reconstruction of mT^+^ and mG^+^ tumor clones using WinSurf software version 4.3 was based on consecutive sections encompassing the entire left lobe in a 12-month-old mutant mouse.

### Data availability

The data generated in this study are available upon request from the corresponding author.

## Results

### Restricted *Braf*^*CA*^ activation due to Cre leakage reproduces tumorigenesis of PTC in a preserved thyroid tissue microenvironment

**Figure 1:**
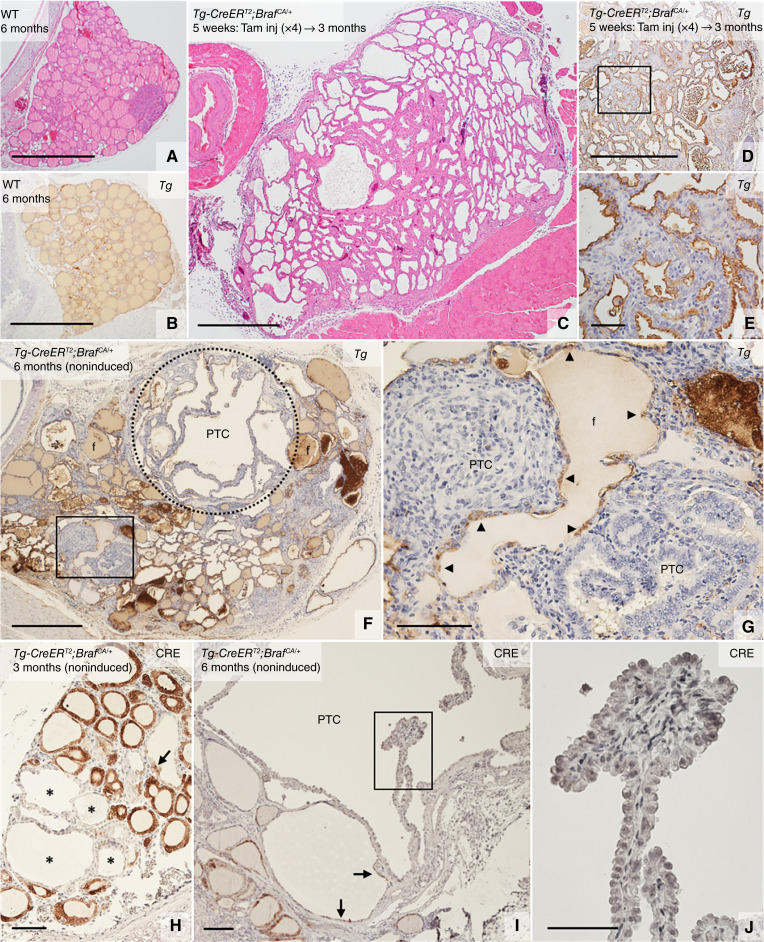
Comparison of tamoxifen-induced and noninduced thyroid tumorigenesis in *Tg-CreER*^*T2*^;*Braf*^*CA/+*^ mice. Representative images of histology and IHC staining of *Tg* and CRE recombinase. **A** and **B,** Thyroid lobe of wild-type (WT) animals. Normal follicular tissue (**A**) and uniform accumulation of *Tg* in follicle lumina (**B**). **C–E,** General neoplastic changes in the thyroid of *Tg-CreER*^*T2*^;*Braf*^*CA/+*^ mice injected with Tam after weaning. Gross lobe enlargement (**A**) and loss of *Tg* expression in cells (**D** and **E**); **E** shows the high power of the boxed area in **D**. Of note, the follicle lumina is largely empty of *Tg*. **F** and **G,** Heterogeneous neoplastic alterations due to spontaneous *Braf*^*CA*^ activation in *Tg-CreER*^*T2*^;*Braf*^*CA/+*^ mice devoid of Tam injections. Loss of *Tg* expression in neoplastic cells and preserved *Tg* expression in nonneoplastic follicles (**F** and **G**). **F** shows the high power of the boxed area in **G**. Arrowheads indicate *Tg*^+^ cells. **H–J,** Loss of CRE expression in neoplastic cells; **J** shows the high power of the boxed area in **I**. Asterisks (in **H**) mark CRE-negative neoplastic follicles. Arrows (in **H** and **I**) indicate residual CRE^+^ cells. Tam, tamoxifen; f, follicle; inj, injection. Bars: 500 (**A–D** and **F**), 100 (**G–I**), and 50 (**E** and **J**) μm.

As previously established (19–21), induced activation of a mutant *Braf* allele targeted to the thyroid gives rise to a generally high proliferative response affecting most if not all thyroid cells in the majority of the follicles, leading to a pronounced enlargement of the gland and eventually transformation to PTC (Fig. 1A and C). Featured by downregulation of *Tg* expression (Fig. 1B, D and E), such global oncogenic activation is accompanied by loss of thyroid differentiation and a severely hypothyroid state (21, 22). Importantly, in this mouse model, thyroid-stimulating hormone (TSH) is initially required for BRAF-induced tumorigenesis (20), presumably related to the hyperplastic surge imposed on the thyroid by the much-elevated circulating TSH levels. By contrast, a more restricted growth response is evident in the absence of tamoxifen induction (from here on referred to as noninduced conditions) due to stochastic *Braf*^*CA*^ activation in but a few cells at the time, which allows modeling of sporadic tumor development with initially most follicles being unaffected and systemic thyroid function maintained (Fig. 1F and G). By this simplified strategy of oncogene activation, which was first reported in 2011 (21), each one of the multifocal tumors likely arises from a single neoplastic follicle. Moreover, although most neoplastic foci are postnatally initiated, tumor growth progresses slowly, signified by macroscopic tumors that do not appear in noninduced mutant mice until 6 to 12 months of age (10).

### Loss of Cre driver expression in Braf mutant thyroid cells prevents reporter gene activation and enables tracing of tumorigenesis of different clonality

**Figure 2:**
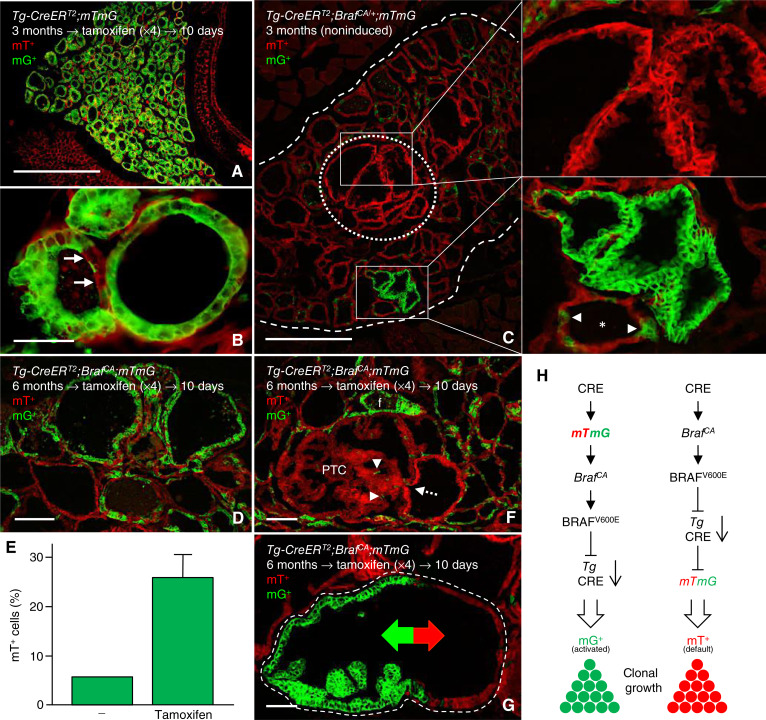
Clonal tracing of thyroid tumor development in noninduced *Tg-CreER*^*T2*^;*Braf*^*CA/+*^;*mTmG* mice. Reporter gene activation designated by switch from mTomato (mT^+^) to mGFP (mG^+^) expression was monitored by fluorescence microscopy. Induced Cre-mediated recombination served as controls for comparison. **A** and **B,** Normal thyroid tissue, overview (**A**) and high power (**B**), after tamoxifen treatment of control mice devoid of *Braf*^*CA*^. Arrows indicate scarce presence of mT^+^ cells escaping induced *mTmG* activation. **C,** Clusters of enlarged/neoplastic follicles exclusively containing mT^+^ (encircled) or mG^+^ cells in a *Braf* mutant mouse devoid of tamoxifen induction. Boxed areas are shown to the right for magnification. Arrowheads indicate single mG^+^ cells present in an adjacent normal follicle (asterisk). **D** and **E,** Widespread induction of mG^+^ thyroid cells in tamoxifen-injected mutant mice; representative image (**D**) and quantitation (*n* = 3; **E**). **F** and **G,** Escape from tamoxifen-induced reporter gene activation in neoplastic follicles and papillary tumor. Arrow (in **F**) indicates origin of tumor derived from a mT^+^ follicle; arrowheads indicate sparse mG^+^ activation among tumor cells. Colored arrows (in **G**) demarcate dual clonal origin of neoplastic follicle (encircled) distinguished by the presence or absence of reporter gene activation. **H,** Predicted labeling of tumor cell progeny depending on whether *mTmG* is stochastically activated before *Braf*^*CA*^ activation (to the left) or hypothetically in cells in which *Braf*^*CA*^ is already activated and the resulting downregulation of the *Cre* driver (the *Tg* promoter) prevents further Cre-mediated recombination, i.e., of the reporter gene (to the right). Bars: 500 (**A** and **C**), 100 (**D** and **F**) 50 (**G**), and 25 (**B**) μm.

As long as constitutive activation of mutant BRAF prevails, thyroid-specific gene expression is suppressed (22). Hence, using *Tg* as *Cre* drivers to accomplish B*raf*^*CA*^ activation implies that Cre downregulates along with BRAFV600E-induced thyroid dedifferentiation. In the sporadic PTC mouse model, this effect is signified by maintained CRE immunoreactivity in nonmutant follicular cells and loss of Cre expression in virtually all cells of neoplastic follicles (Fig. 1H) and tumors (Fig. 1I and J). As previously demonstrated (10), this causation restrains reporter gene activation to account only those cells that do not yet express mutant BRAF. This indicates that it should be possible to monitor propagation of a tumor clone from the very start of *Braf*^*CA*^ activation provided that *mTmG* is activated before or simultaneously with the oncogene in the ancestral mutant cell. Confirming this notion, in control tracing experiments on *Tg-CreERT2*;*mTmG* mice with normal thyroid structure and function, it is evident that nearly all follicular cells respond to induced Cre activation and become green fluorescent (Fig. 2A and B). By contrast, in 3-month-old noninduced *Tg-CreER*^*T2*^;*Braf*^*CA/+*^;*mTmG* mutant mice, reporter gene activation is restricted to truly neoplastic follicles in which all cells already express GFP (due to prior activation) and scattered mG^+^ cells present in seemingly normal follicles (Fig. 2C and insets).

It is noteworthy, however, that many enlarged/neoplastic follicles are entirely devoid of mG^+^ cells, suggesting they represent BRAF mutant clones failing to activate Cre after *Braf*^*CA*^ expression is turned on and the cells start to dedifferentiate. Indeed, as an illustration of this assumption – and proof-of-concept of the working model of clonal tracing deduced by the differentiation status of targeted cells – the number of mG^+^ cells increased only to a limited extent after induced Cre activation by tamoxifen in adult mutants (Fig. 2D and E). The presence of nonresponding cells is thus in all probability explained by downregulation of Cre because of constitutive activation of the MAPK pathway mediated by BRAFV600E. Accordingly, response failure to induction indicates that many *Braf* mutant cells are stationary or only slowly proliferate, probably involving oncogene-induced senescence (23, 24). As a consequence, although spontaneous Cre-mediated *Braf*^*CA*^ activation is widespread and accumulates over time, signs of overt tumor development are limited to a handful loci per lobe which enables tracing of clonality with high accuracy and resolution. For example, oligoclonal tumorigenesis with different growth patterns of individual clones is evident by dual labeling (mG^+^ vs. mT^+^) of neoplastic follicles in which the entire mT^+^ epithelial domain is unresponsiveness to tamoxifen (Fig. 2F and G; Supplementary Fig. S1A–S1H). Taken collectively, these observations infer depending on whether the reporter gene is activated along with the oncogene or not at all, and provided dedifferentiation is not reversed, e.g., by BRAF kinase inhibition (10, 22), any tumor clone expanding from a BRAF mutant cell will be permanently labeled green or red fluorescent (Fig. 2H).

### Braf mutant clones with shared follicle origin adopt identical and spatially integrated papillary tumor growth patterns

**Figure 3:**
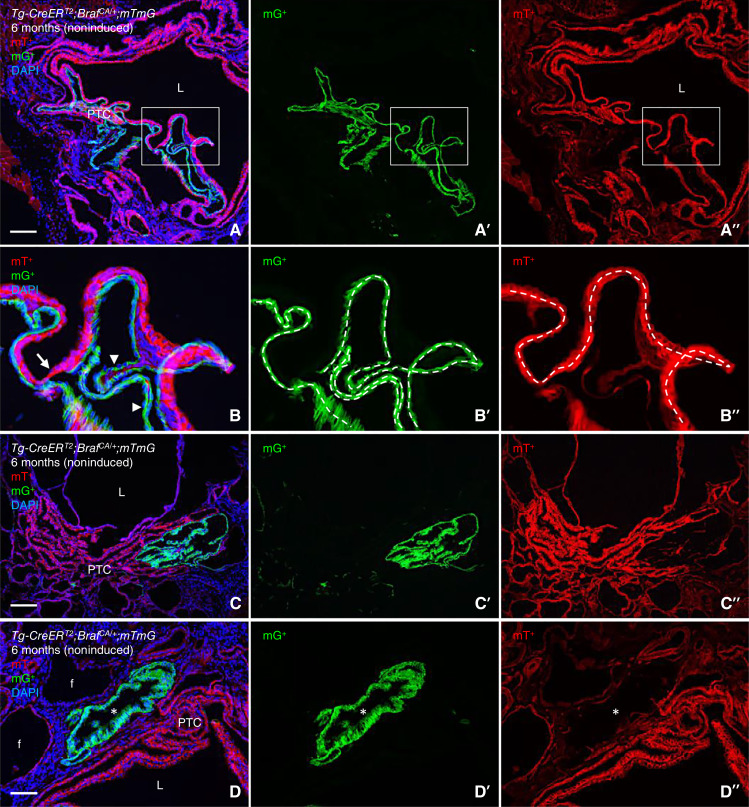
Oligoclonal growth of BRAF-induced thyroid tumors revealed by clonal tracing. Images obtained from 6-month-old noninduced *Tg-CreER*^*T2*^;*Braf*^*CA/+*^;*mTmG* mice. Separate green (**A′–D′**) and red channels (**A″–D″**) are shown for clarity. **A** and **B,** Coordinated growth of contiguous mT^+^ and mG^+^ tumor clones. **B–B″** show the high power of the boxed areas in **A–A″**. Indistinguishable growth patterns were noted except for minor mT^+^ (arrow) and mG^+^ (arrowheads) epithelial segments (outlined in **B′** and **B″** for comparison). **C,** Similar papillary growth patterns of adjoining mT^+^ and mG^+^ tumor clones. **D,** Dissimilar growth patterns of nearby but not connecting mT^+^ and mG^+^ tumor clones. Asterisks indicate cluster of neoplastic follicles. DAPI stained nuclei (in **A–D**). f, follicle; L, lumen. Bars: 100 (**A**, **C**, and **D**) μm.

The mutually exclusive activation or silencing of the *mTmG* reporter further revealed that some mG^+^ and mT^+^ tumor clones form nearly perfectly aligned epithelial sheets separated only by a thin intervening layer of stromal cells, consistent with an oligoclonal mechanism of coordinated papillary growth (Fig. 3A–A″ and B–B″). It is likely that the appearance of such microcarcinomas – developed by extensive convolution of the neoplastic epithelium of a single, much-enlarged follicle – would be regarded as monoclonal were it not for the dual clonal origin uncovered in this study by lineage tracing. Other compound lesions consisting of not fully merged mT^+^ and mG^+^ tumor domains show similar but not identical growth patterns (Fig. 3C–C″). Observations of adjacent clone size differences suggest that clone properties might differ regarding intrinsic growth rates or alternatively they evolved asynchronously and by chance happened to develop abreast (Fig. 3D–D″). We cannot exclude that BRAFV600E-induced mouse thyroid tumors may develop from only one ancestral mutant cell, consistent with the current concept of a monoclonal origin of human PTC (25). However, from this and previous findings (10), it is evident that noninduced *Braf*^*CA*^ activation generates oligoclonal tumors and that spatial proximity of BRAF mutant clones – proven by lineage tracing to have distinct ancestral cell origins – triggers the coordinated formation of true papillae.

### Clonal transition from papillary to follicular tumor phenotype correlates with abundance of stromal tissue and infiltrative growth

**Figure 4:**
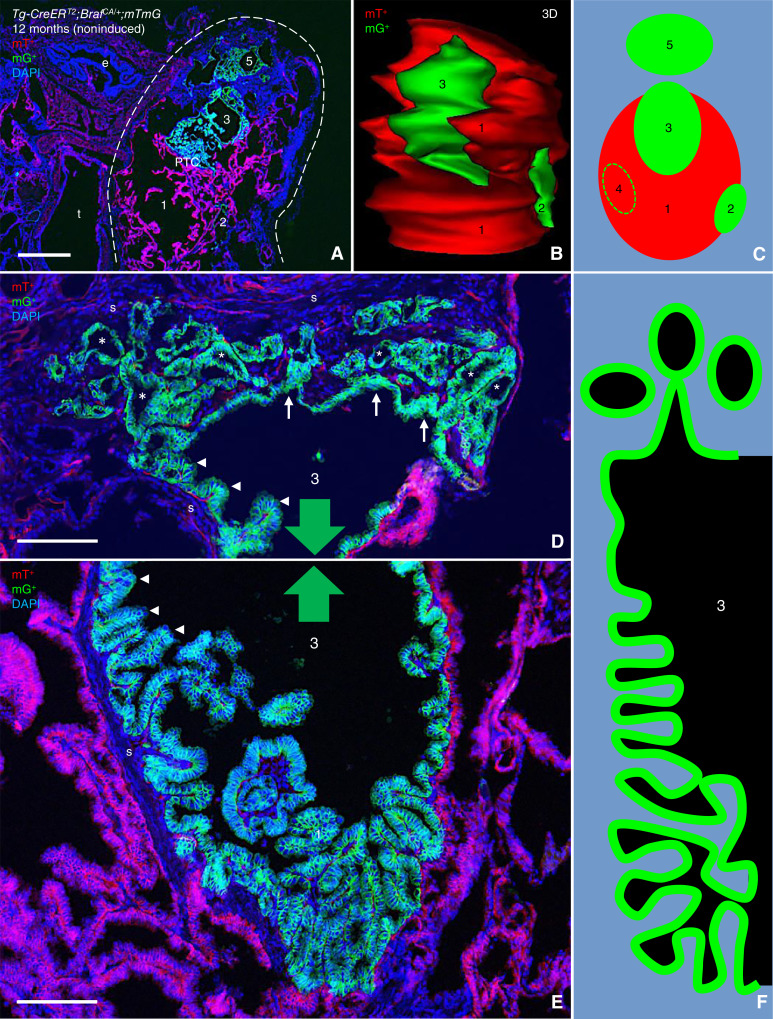
Papillary–follicular transition in *Braf*^*CA*^-induced PTC. Advanced tumor stage of a 12-month-old noninduced *Tg-CreER*^*T2*^;*Braf*^*CA/+*^;*mTmG* mouse. Both thyroid lobes were serially sectioned from pole to pole for comprehensive evaluation. **A–C,** Overview of tumors present in the left thyroid lobe (encircled); transverse tissue section (**A**), 3D reconstruction (**B**), and schematic representation (**C**) of the predominant mT^+^ tumor clone (1) and associated mG^+^ clones (2–5). **D–F,** Distal/dorsal domain (**D**), proximal/ventral domain (**E**), and schematic representation (**F**) of mG^+^ clone 3. Arrows and arrowheads indicate outward evaginations and inward papillary formations, respectively, in consecutive segments of the same neoplastic epithelium of a giant follicle. Asterisks indicate follicular differentiation of emigrating mG^+^ tumor cells. Directions of continuity of clone 3 are indicated by green-colored arrows (in **D** and **E**). DAPI stained nuclei (in **A**, **D** and **E**). e, esophagus; t, trachea; s, stromal tissue of tumor. Bars: 500 (**A**) and 100 (**D** and **E**) μm.

From previous studies, we know that thyroid tumor growth accelerates between 6 and 12 months in the present model of sporadic PTC development (10). To elucidate the extent of clonal interactions in more advanced tumor stages, we conducted long-term lineage tracing experiments and serially sectioned from pole to pole the much enlarged thyroid of 12-month-old noninduced *TgCreER*^*T2*^;*Braf*^*CA/+*^;*mTmG* mice (*n* = 4) for comprehensive evaluation microscopically. Remarkably, we found salient mG^+^ tumor foci in only one of eight lobes (Fig. 4A); the reason for clonal selection in favor of mT^+^ tumors is probably a growth advantage of cells being transformed postnatally, therefore possessing less chance of spontaneously activating the reporter gene prior to *Braf*^*CA*^ activation (10). 3D reconstruction of the lobe of interest revealed a centrally located large mT^+^ tumor numbered “1” with three closely associated smaller mG^+^ components numbered “2 to 4” (Fig. 4B; image obtained from a ventrolateral projection of 3D animation; see Supplementary Movie S1). An additional much larger mG^+^ tumor clone numbered “5” appeared dorsally at some distance from the others (depicted in Fig. 4C).

With exception of clone 5 (to be described in a following paragraph), all enumerated tumor clones 1 to 4 showed a characteristic papillary growth pattern. However, clone 3 stands out as it is further distinguished by two opposite portions with distinctly different morphologies—on one side a typical papillary component facing clone 1 and on the other side a conspicuous follicular component that seemingly is buried in abundance of connective tissue (Fig. 4D and E). The papillary domain of the neoplastic epithelium thus projects into the shared lumen, whereas the follicular domain buckles outward and grows infiltratively by forming numerous small follicles (Fig. 4D). Moreover, the numerous epithelial infoldings gradually diminish in number and height toward the follicular portion with a transition zone consisting of a flat epithelial segment in between (Fig. 4F, depicted from Fig. 4D and E).

From this, we conclude that Braf mutant thyroid cells are capable of transforming from a papillary to a follicular tumor phenotype designated by clonal plasticity. A possible involvement of a subclonal event, accomplished by additional somatic mutations which are rare and inconsistent in noninduced *TgCreER*^*T2*^;*Braf*^*CA/+*^;*mTmG* mice of the same age (10), cannot be excluded. However, it would unlikely be a single cause of gradual morphologic changes correlating with the gradient of nearby stromal cell appearance.

### Mutant Braf rarely triggers *de novo* FTC development in mice

**Figure 5:**
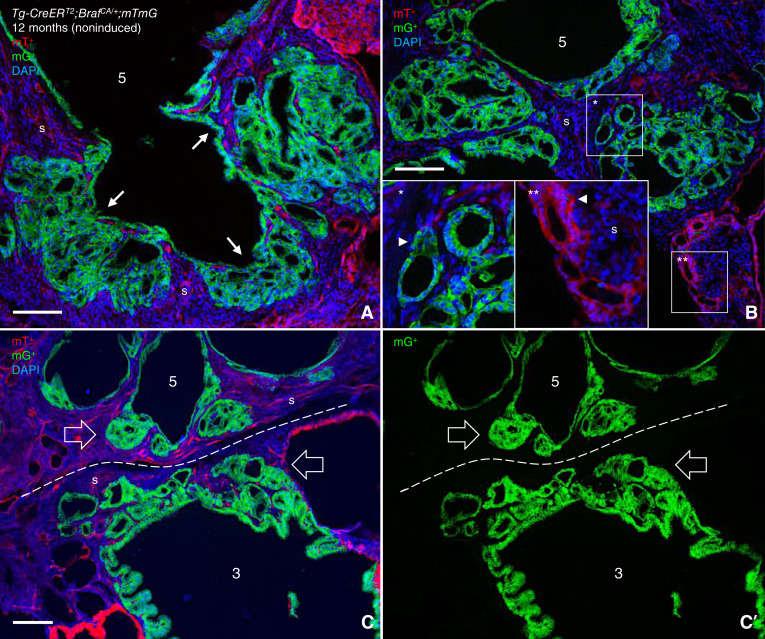
Clonal folliculogenesis in response to *Braf*^*CA*^ activation. Images from serially sectioned thyroid specimen in the same *Tg-CreER*^*T2*^;*Braf*^*CA/+*^;*mTmG* mouse in which individual tumor clones are identified and numbered as outlined in Fig. 4. **A–C,** Follicular phenotype of mT^+^ tumor clone 5, from selected consecutive sections of which the entire series is provided in Supplementary Fig. S2. Arrows (in **A**) indicate predilection sites of outward directed migration of folliculogenic tumor cells. Insets (in **B**, identified by asterisks) show the high power of the boxed areas, with arrowheads indicating the asymmetric shape of neoplastic mG^+^ and mT^+^ follicles (of different clonal origins). Open arrows (in **C**) indicate clusters of G^+^ neoplastic follicles derived from either of clone 3 or 5; **C′** shows only the green channel for clarity of morphology, including the intervening stromal space (dashed lines) that separates the two clones. DAPI stained nuclei (in **A–C**). S, stromal tissue of tumor. Bars: 100 μm.

The largest mG^+^ tumor referring to clone 5 differed much from the other lesions as it did not display any typical papillary formation but consisted of multiple cysts with thickening of the wall at regular intervals comprising multifocal expansions of neoplastic follicles notably embedded in stromal tissue (Fig. 5A and B). Serial sectioning revealed that a portion of clone 5 is facing the follicular domain of clone 3, although a narrowing layer of stromal cells separates the two mG^+^ tumors in all instances, confirming they are clonally distinct (Fig. 5C and C′; Supplementary Fig. S2A–S2G). Notably, closely located mT^+^ cells also form follicle-like structures with similar appearance and stromal embedding as mG^+^ follicles [Fig. 5B (insets)].

Considering the overall preponderance of tumors with the papillary phenotype in *TgCreER*^*T2*^;*Braf*^*CA/+*^ mice, the appearance of follicular-like tumors of different clonality in a limited part of the thyroid lobe is hardly coincidental but rather suggests that the immediate tumor microenvironment provides some trophic factor(s) that promote follicle formation, possibly at the expense of papillogenesis, in any adjacent tumor clone. Observation of a follicular pattern of infiltrative growth in a mouse model of BRAFV600E-induced PTC is intriguing in view of previous notions that BRAF largely predominates over RAS as a driver mutation in follicular-variant PTC tumors with invasive properties in humans (6).

## Discussion

A well-known characteristic of PTC is the markedly variable tumor growth pattern which by routine histologic examination forms the basis of classification of PTC subtypes (1). However, in human PTC, an association between tumor subtype and genetic lesion is only rarely encountered, e.g., *RET*/*PTC3* rearrangement is more frequent in the solid variant of PTC being overrepresented in radiation-induced thyroid cancer (26, 27). Because most cases of sporadic PTC irrespective of the histopathology share BRAFV600E as an oncogenic driver and secondary mutations of pathogenic significance, e.g., of *TERT* and *TP53*, usually are late events implicated in tumor progression, it is rationale to infer the existence of yet unknown factors that likely contribute to the development of different PTC tumor phenotypes.

One reason to the sparse knowledge of mechanisms that govern PTC morphogenesis and tumor heterogeneity is the lack of suitable experimental models that allow monitoring of tumor development over longer periods of time and at least to some extent would reproduce the evolution of PTC in humans. Transgenic mouse models of thyroid cancer usually use a ubiquitously expressed thyroid gene (e.g., *Tg* or *Tpo*) as a Cre driver for targeted oncogene activation, which inevitably involves the majority of cells and thus deviates much from the sporadic and presumed monoclonal nature of PTC tumorigenesis (25). Another disadvantage of induced oncogenic activation, e.g., by tamoxifen is that the enforced general neoplastic growth will deteriorate the normal thyroid histoarchitecture and render mice severely hypothyroid, leading to markedly increased TSH blood levels (19, 21). Although TSH is required for tumor initiation and to overcome oncogene-induced senescence (20, 23), a complicating factor of supraphysiologic TSH is goitrogenesis, which makes it difficult to distinguish neoplastic from reactive growth and confound the possibility to determine and monitor tumor clonality. By contrast, spontaneous thyroid tumorigenesis in Braf mutant mice achieved by omitting induction, as originally reported by the McMahon group (21), occurs in euthyroid conditions and offers a more realistic means of investigating tumor development in a preserved thyroid tissue microenvironment (10). By ultrasensitive sequencing of minute amounts of the activated Braf mutant allele, it is estimated that in noninduced conditions, transformed cells expressing the *Braf* mutant allele comprise less than 1% of the total thyroid cell number in young adult *CreER*^*T2*^;*Braf*^*CA/+*^ mice (28).

Our lineage tracing experiments identified two principally different clonal growth patterns, in most cases papillary and less frequently follicular, of individual tumor lesions. As previously elucidated (10) and confirmed in the present study, fully developed thyroid papillae may have a labyrinthine appearance but are essentially based on planar growth and excessive folding of the neoplastic epithelium, primarily resulting from lateral expansion due to oriented cell divisions perpendicular to the apical–basal axis of cells that originally derive from a single follicle. Thus, papillary morphogenesis resembles at least initially the general pattern of epithelial folding governed by mechanical forces transduced into nonuniform geometric deformations (29).

Elucidated in fruit fly, epithelial folding seems to be a tightly regulated process that requires epithelial cell shape changes involving shifted position of the adherens junctions (30) and differential growth rates that predict fold positioning (31). Whether carcinoma cells might adopt, or hijack for their uncontrolled expansion, any basic mechanism of natural epithelial folding is unknown. The fact that Braf mutant cells are capable of forming papillae although the originating follicle displays plenty of luminal space argues that papillary growth is not merely a consequence of mechanical stress due to cell crowding. It is also evident from the present study that some oligoclonal follicles representing early papillary lesions involve one but not the other clone. Moreover, enlarged follicles harboring BRAF mutant cells (identified by loss of Cre expression due to dedifferentiation) may alternatively consist of an epithelial layer that does not, or only to a minor extent, convolute. Collectively, these observations indicate that thyroid follicular cells possess a markedly heterogeneous responsiveness to oncogenic activation by mutant Braf, which probably is inherent to their different abilities to proliferate and eventually commence papillary growth.

Previous studies have shown that presence of papillae is a major diagnostic criterion differentiating PTC from noninvasive follicular neoplasia with papillary-like nuclear features and also that the number of true papillae (i.e., with a fibrovascular core) correlates with lymph node metastasizing capacity (32). Elucidating mechanisms of BRAF-induced papillogenesis and the basis of heterogeneous response to mutant BRAF are therefore of potential clinical value. Thyroid follicles are naturally heterogeneous regarding size and the contributing number of epithelial cells, which correlates to different functional activity, e.g., in iodide uptake and intrathyroidal iodine metabolism (11, 13, 33–35). There is also a great variation in gene expression among cells within a single follicle, suggesting that thyroid cells might exert different roles and cooperate in the overall production of thyroid hormone and maintenance of systemic thyroid function (36–39). Earlier work in rodents infers that the cell renewal rate in the adult thyroid is very low for as long as thyroid growth is not provoked, e.g., by exposure to goitrogens, and that mitogenesis is restricted to subpopulations of growth-prone follicular cells (11, 40). Although all thyroid cells are capable of entering the cell cycle [e.g., this occurs naturally in early thyroid development (41, 42) and in primary cultured thyroid cells, e.g., after epidermal growth factor stimulation (43)], it is conceivable that cells possess different thresholds responding to a given concentration or gradient of a growth stimulus. Indeed, mitogen sensitivity is heterogeneous among thyroid cells depending on the level of TSH stimulation via the cyclic AMP pathway (44, 45).

Whether such functional heterogeneities might influence the response to oncogenic activation has not been investigated in much detail. However, BRAFV600E-induced neoplastic growth is obviously less efficient in adult mice than postnatally (46), which is consistent with a higher radiosensitivity and risk of developing thyroid cancer after radioiodine (RAI) exposure of the growing gland in children (47). Analogous to the pathogenesis of multinodular goiter (12, 33), we recently proposed that the natural follicle heterogeneity is an independent determinant of thyroid tumor development and, moreover, that follicle maturity and the interplay of mutant and nonmutant cells matter whether a tumor clone is successfully propagated or not (10). In support of this model, high iodine content associated with inactive follicles *in vivo* has been shown to attenuate oncogenic activation in cultured thyroid cells (48, 49).

Folliculogenesis as typically occurs in thyroid regeneration, benign thyroid hyperplasia, and follicular adenoma is recapitulated in FTC and the follicular variant of PTC. In principle, the follicular growth pattern of tumors is not fundamentally different from neoformation of follicles during thyroid development (50) or experimentally in *in vitro*–reconstituted follicles challenged with growth factors while embedded in collagen-based extracellular matrix (51) and by embryonic stem cells committed to a thyroid fate (52). In the adult gland, folliculogenesis arguably occurs by displacement of cells from the mother follicle and their reassociation into satellite microfollicles (12, 53). However, it is noteworthy that although generation of new follicles requires thyroid cell renewal, the morphogenetic process initially comprises cell migration (outward viz the originating follicle) that is uncoupled from cell proliferation (54). In the present study, as revealed by reporter tracing of clonality, we observed generation of neoplastic follicles in advanced-stage tumor lesions. Moreover, the tumor stroma was infiltrated by neoplastic follicles of different clonal origin, suggesting that local factors presumably elicited by the prime folliculogenic tumor clone might promote folliculogenesis amongst the others. This is of interest considering that the precise mechanism that triggers normal thyroid cells to reorganize into follicles it is yet unclear. TSH is dispensable for *de novo* follicle formation in fetal thyroid development (55). Nkx2-1 and Pax8 are required for thyroid differentiation of both embryonic and pluripotent stem cells (52, 56), but in the embryo, these key transcription factors are expressed long before follicles appear, consistent with a permissive rather than inductive role (57). The fact that mutant BRAF activation in mouse models strongly suppress the expression of thyroid differentiation genes (10, 21, 22) but promotes clonal development of neoplastic follicles (this study) is consistent with previous notions that thyroid function and folliculogenesis are separately regulated across species. Indeed, development of the follicular thyroid relies on evolutionary conserved mechanisms that can be traced back to the divergence of vertebrate and nonvertebrate chordates whereas the ability to iodinate and produce thyroid hormone production is even more ancient (58–60).

It is likewise unknown which mechanisms differ natural from neoplastic folliculogenesis with the potential and capacity of mutant thyroid cells to metastasize and reorganize into follicles in other organ sites. Notably, distant metastasis is a typical feature of FTC but is much less frequent in patients with PTC (61). Findings of RAS- and BRAF-like molecular signatures in PTC subtypes defined by transcriptome profiling (62), which also distinguish follicular variants of PTC with different tendencies of regional nodal disease (6), suggest that mutation identity (*RAS* vs. *BRAF*) is more important for tumor invasiveness and spreading rather than determining the histologic tumor growth pattern. That development of one or the other tumor type is not exclusively linked to a specific mutation is supported by reports of *RAS* driver mutations in BRAFV600E-negative follicular-variant PTC (6, 7, 63) and *BRAF* mutations in rare cases of FTC (8, 9). However, regardless these exceptions, the archetypical growth pattern of FTC and PTC is collectively strongly associated with *RAS* and BRAF mutations, respectively. It is possible that the lower magnitude and reversibility of constitutive MAPK pathway activation in cells with RAS-like transcriptional output, which correlates with preserved expression of thyroid differentiation genes (61), might play a role. The fact that Ras, unlike its downstream signaling partner Raf, associates with the plasma membrane and additionally activates other signaling pathways, which makes drug development targeting of Ras a more challenging caveat (64, 65), provides a logical basis for explaining differing biological effects and outcomes of *RAS* versus *BRAF* driver mutations in the thyroid. Nonetheless, although *RAS* mutant metastatic thyroid cancer shows better responsiveness to RAI therapy than *BRAF* mutant tumors with similar clinical characteristics, both driver mutations are encountered among RAI responders and nonresponders (66). It is thus yet unproven whether and how dimeric RAF activation by RAS oncoproteins and monomeric signaling of BRAFV600E, which constitute the basis of the difference in MAPK signaling output, might differently rewire the growth mode of mutant thyroid cells promoting either follicular or papillary tumorigenesis.

**Figure 6:**
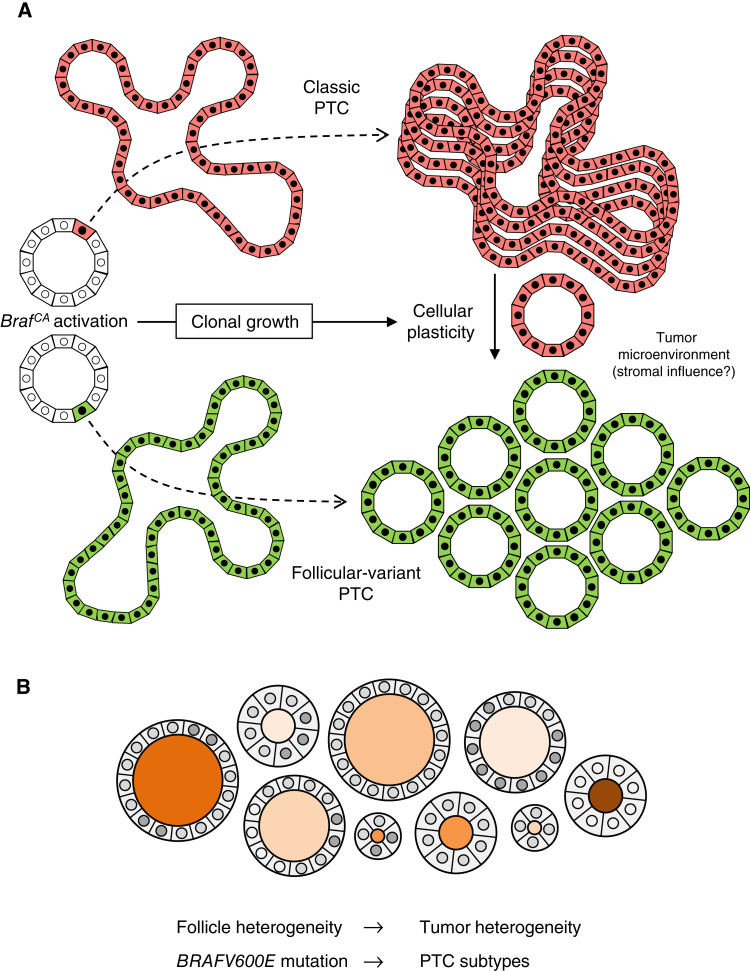
Modeling the evolution of BRAFV600E-driven PTC subtypes based on the present findings. **A,** Differential tumor growth patterns evolving into classic PTC (top) or follicular-variant PTC (bottom) supposedly depend on intrinsic features of the originating follicle, tumor cell plasticity, and microenvironmental factors. **B,** Follicle heterogeneity, confirmed experimentally by us and others, likely forms the basis of heterogeneous tumor development in the mouse thyroid. Being a conspicuous feature also of the human thyroid gland, follicle heterogeneity might conceivably contribute to divergent tumor histogenesis of PTC subtypes irrespective of driver mutation identity.

In summary, in this study, we show by lineage tracing of multifocal thyroid tumor development in noninduced *TgCreER*^*T2*^;*Braf*^*CA/+*^;*mTmG* mice that follicular cells possess a remarkable plasticity of neoplastic growth comprising papillary and, less frequently, follicular tumor phenotypes, which are distinguished and possibly determined by the follicle origin of the mutant clone (Fig. 6A). Pleomorphism of BRAFV600E-driven thyroid cancer as evident clinically by diagnosis of the various PTC subtypes might thus arise at least partly depending on intrinsically heterogeneous properties of thyroid follicles that stochastically harbor a mutant cell (Fig. 6B). This model infers that ancestral tumor cell-of-origin features are transmitted to the progeny and influence PTC histogenesis, similar to previously established concepts for the pathogenesis of multinodular goiter (33) and thyroid nodules (67). Additionally, clonal transition from papillary to follicular growth patterns may occur possibly elicited by the tumor cells themselves or another tumor clone that is intrinsically folliculogenic and might transmit this ability to their neighbors by modifying the immediate tumor microenvironment (Fig. 6B). Whether a putative reverse transition from follicular to papillary tumorigenesis also exists is more difficult to comprehend because DTCs conceptually originate and develop from follicles. Tracing RAS-driven thyroid tumor development would probably serve a better model to elucidate whether and how RAS mutant tumor cells might switch from follicular to papillary growth mode and generate follicular variant PTC as encountered in patients (6). Molecular mechanisms decisive for oncogene-transformed thyroid cells adopting papillary or follicular morphogenetic traits, which basically rely on different epithelial folding mechanics and the direction of cell divisions that shape natural epithelia (29, 68), remain open questions.

## Supplementary Material

Movie 1 animation

Figure S1 IF images Figure S1. Clonal tracing of sporadically developed BRAF mutant neoplasia in mouse thyroid.

Figure S2 IF images Figure S2. Clonal tracing of BRAF mutant thyroid carcinoma that displays a follicular tumor phenotype.

## References

[1] Baloch ZW , Asa SL, Barletta JA, Ghossein RA, Juhlin CC, Jung CK, . Overview of the 2022 WHO classification of thyroid neoplasms. Endocr Pathol 2022;33:27–63. 35288841 10.1007/s12022-022-09707-3

[2] Fagin JA , Krishnamoorthy GP, Landa I. Pathogenesis of cancers derived from thyroid follicular cells. Nat Rev Cancer 2023;23:631–50. 37438605 10.1038/s41568-023-00598-y PMC10763075

[3] Scheffel RS , Dora JM, Maia AL. BRAF mutations in thyroid cancer. Curr Opin Oncol 2022;34:9–18. 34636352 10.1097/CCO.0000000000000797

[4] Howell GM , Hodak SP, Yip L. RAS mutations in thyroid cancer. Oncologist 2013;18:926–32. 23873720 10.1634/theoncologist.2013-0072 PMC3755930

[5] Hernandez-Prera JC , Wenig BM. RAS-mutant follicular thyroid tumors: a continuous challenge for pathologists. Endocr Pathol 2024;35:167–84. 38888731 10.1007/s12022-024-09812-5

[6] Rivera M , Ricarte-Filho J, Knauf J, Shaha A, Tuttle M, Fagin JA, . Molecular genotyping of papillary thyroid carcinoma follicular variant according to its histological subtypes (encapsulated vs infiltrative) reveals distinct BRAF and RAS mutation patterns. Mod Pathol 2010;23:1191–200. 20526288 10.1038/modpathol.2010.112 PMC4573468

[7] Di Cristofaro J , Marcy M, Vasko V, Sebag F, Fakhry N, Wynford-Thomas D, . Molecular genetic study comparing follicular variant versus classic papillary thyroid carcinomas: association of N-ras mutation in codon 61 with follicular variant. Hum Pathol 2006;37:824–30. 16784981 10.1016/j.humpath.2006.01.030

[8] Pennelli G , Vianello F, Barollo S, Pezzani R, Merante Boschin I, Pelizzo MR, . BRAF(K601E) mutation in a patient with a follicular thyroid carcinoma. Thyroid 2011;21:1393–6. 22136270 10.1089/thy.2011.0120

[9] Repaci A , Salituro N, Vicennati V, Monari F, Cavicchi O, de Biase D, . Unexpected widespread bone metastases from a BRAF K601N mutated follicular thyroid carcinoma within a previously resected multinodular goiter. Endocr Pathol 2022;33:519–24. 34843063 10.1007/s12022-021-09698-7

[10] Schoultz E , Johansson E, Moccia C, Jakubikova I, Ravi N, Liang S, . Tissue architecture delineates field cancerization in BRAFV600E-induced tumor development. Dis Model Mech 2022;15:dmm048887. 34379110 10.1242/dmm.048887 PMC8380047

[11] Peter HJ , Studer H, Forster R, Gerber H. The pathogenesis of “hot” and “cold” follicles in multinodular goiters. J Clin Endocrinol Metab 1982;55:941–6. 7119088 10.1210/jcem-55-5-941

[12] Peter HJ , Gerber H, Studer H, Smeds S. Pathogenesis of heterogeneity in human multinodular goiter. A study on growth and function of thyroid tissue transplanted onto nude mice. J Clin Invest 1985;76:1992–2002. 4056062 10.1172/JCI112199 PMC424262

[13] Many MC , Denef JF, Hamudi S, Haumont S. Increased follicular heterogeneity in experimental colloid goiter produced by refeeding iodine excess after thyroid hyperplasia. Endocrinology 1986;118:637–44. 3943488 10.1210/endo-118-2-637

[14] Thomas GA , Williams D, Williams ED. The demonstration of tissue clonality by X-linked enzyme histochemistry. J Pathol 1988;155:101–8. 3164772 10.1002/path.1711550205

[15] Ahuja S , Schiller S, Ernst H. An autoradiography study of postoperatively labelled thyroid tissue and iodine storage. Eur J Nucl Med 1991;18:791–5. 1743201 10.1007/BF00175056

[16] Undeutsch H , Löf C, Offermanns S, Kero J. A mouse model with tamoxifen-inducible thyrocyte-specific cre recombinase activity. Genesis 2014;52:333–40. 24395757 10.1002/dvg.22740

[17] Dankort D , Filenova E, Collado M, Serrano M, Jones K, McMahon M. A new mouse model to explore the initiation, progression, and therapy of BRAFV600E-induced lung tumors. Genes Dev 2007;21:379–84. 17299132 10.1101/gad.1516407 PMC1804325

[18] Muzumdar MD , Tasic B, Miyamichi K, Li L, Luo L. A global double-fluorescent Cre reporter mouse. Genesis 2007;45:593–605. 17868096 10.1002/dvg.20335

[19] Knauf JA , Ma X, Smith EP, Zhang L, Mitsutake N, Liao X-H, . Targeted expression of BRAFV600E in thyroid cells of transgenic mice results in papillary thyroid cancers that undergo dedifferentiation. Cancer Res 2005;65:4238–45. 15899815 10.1158/0008-5472.CAN-05-0047

[20] Franco AT , Malaguarnera R, Refetoff S, Liao X-H, Lundsmith E, Kimura S, . Thyrotrophin receptor signaling dependence of Braf-induced thyroid tumor initiation in mice. Proc Natl Acad Sci U S A 2011;108:1615–20. 21220306 10.1073/pnas.1015557108 PMC3029699

[21] Charles R-P , Iezza G, Amendola E, Dankort D, McMahon M. Mutationally activated BRAF(V600E) elicits papillary thyroid cancer in the adult mouse. Cancer Res 2011;71:3863–71. 21512141 10.1158/0008-5472.CAN-10-4463 PMC3107361

[22] Chakravarty D , Santos E, Ryder M, Knauf JA, Liao X-H, West BL, . Small-molecule MAPK inhibitors restore radioiodine incorporation in mouse thyroid cancers with conditional BRAF activation. J Clin Invest 2011;121:4700–11. 22105174 10.1172/JCI46382 PMC3225989

[23] Zou M , Baitei EY, Al-Rijjal RA, Parhar RS, Al-Mohanna FA, Kimura S, . TSH overcomes Braf(V600E)-induced senescence to promote tumor progression via downregulation of p53 expression in papillary thyroid cancer. Oncogene 2016;35:1909–18. 26477313 10.1038/onc.2015.253 PMC6310059

[24] Bellelli R , Vitagliano D, Federico G, Marotta P, Tamburrino A, Salerno P, . Oncogene-induced senescence and its evasion in a mouse model of thyroid neoplasia. Mol Cell Endocrinol 2018;460:24–35. 28652169 10.1016/j.mce.2017.06.023 PMC5741508

[25] Ghossein RA , Katabi N, Fagin JA. Immunohistochemical detection of mutated BRAF V600E supports the clonal origin of BRAF-induced thyroid cancers along the spectrum of disease progression. J Clin Endocrinol Metab 2013;98:E1414–21. 23775351 10.1210/jc.2013-1408 PMC6287446

[26] Nikiforov YE , Rowland JM, Bove KE, Monforte-Munoz H, Fagin JA. Distinct pattern of ret oncogene rearrangements in morphological variants of radiation-induced and sporadic thyroid papillary carcinomas in children. Cancer Res 1997;57:1690–4. 9135009

[27] Powell DJ Jr , Russell J, Nibu K, Li G, Rhee E, Liao M, . The RET/PTC3 oncogene: metastatic solid-type papillary carcinomas in murine thyroids. Cancer Res 1998;58:5523–8. 9850089

[28] Schoultz E , Liang S, Carlsson T, Filges S, Ståhlberg A, Fagman H, . Tissue specificity of oncogenic BRAF targeted to lung and thyroid through a shared lineage factor. iScience 2023;26:107071. 37534159 10.1016/j.isci.2023.107071 PMC10391731

[29] Wang Y-C . The origin and the mechanism of mechanical polarity during epithelial folding. Semin Cell Dev Biol 2021;120:94–107. 34059419 10.1016/j.semcdb.2021.05.027

[30] Wang Y-C , Khan Z, Kaschube M, Wieschaus EF. Differential positioning of adherens junctions is associated with initiation of epithelial folding. Nature 2012;484:390–3. 22456706 10.1038/nature10938 PMC3597240

[31] Tozluoǧlu M , Duda M, Kirkland NJ, Barrientos R, Burden JJ, Muñoz JJ, . Planar differential growth rates initiate precise fold positions in complex epithelia. Dev Cell 2019;51:299–312.e4. 31607650 10.1016/j.devcel.2019.09.009 PMC6838681

[32] Xu B , Serrette R, Tuttle RM, Alzumaili B, Ganly I, Katabi N, . How many papillae in conventional papillary carcinoma? A clinical evidence-based pathology study of 235 unifocal encapsulated papillary thyroid carcinomas, with emphasis on the diagnosis of noninvasive follicular thyroid neoplasm with papillary-like nuclear features. Thyroid 2019;29:1792–803. 31452453 10.1089/thy.2019.0328 PMC6918873

[33] Studer H , Peter HJ, Gerber H. Natural heterogeneity of thyroid cells: the basis for understanding thyroid function and nodular goiter growth. Endocr Rev 1989;10:125–35. 2666115 10.1210/edrv-10-2-125

[34] Mestdagh C , Many MC, Halpern S, Briançon C, Fragu P, Denef JF. Correlated autoradiographic and ion-microscopic study of the role of iodine in the formation of “cold” follicles in young and old mice. Cell Tissue Res 1990;260:449–57. 2372804 10.1007/BF00297224

[35] Penel C , Rognoni JB, Bastiani P. Thyroid morphological and functional heterogeneity: impact on iodine secretion. Gen Physiol Biophys 1985;4:55–68. 4029592

[36] Suzuki K , Mori A, Lavaroni S, Miyagi E, Ulianich L, Katoh R, . In vivo expression of thyroid transcription factor-1 RNA and its relation to thyroid function and follicular heterogeneity: identification of follicular thyroglobulin as a feedback suppressor of thyroid transcription factor-1 RNA levels and thyroglobulin synthesis. Thyroid 1999;9:319–31. 10319936 10.1089/thy.1999.9.319

[37] Pohl V , Roger PP, Christophe D, Pattyn G, Vassart G, Dumont JE. Differentiation expression during proliferative activity induced through different pathways: in situ hybridization study of thyroglobulin gene expression in thyroid epithelial cells. J Cell Biol 1990;111:663–72. 2199463 10.1083/jcb.111.2.663 PMC2116189

[38] Pohl V , Abramowicz M, Vassart G, Dumont JE, Roger PP. Thyroperoxidase mRNA in quiescent and proliferating thyroid epithelial cells: expression and subcellular localization studied by in situ hybridization. Eur J Cell Biol 1993;62:94–104. 8269983

[39] Gillotay P , Shankar M, Haerlingen B, Sema Elif E, Pozo-Morales M, Garteizgogeascoa I, . Single-cell transcriptome analysis reveals thyrocyte diversity in the zebrafish thyroid gland. EMBO Rep 2020;21:e50612. 33140917 10.15252/embr.202050612 PMC7726803

[40] Smeds S , Peter HJ, Jörtsö E, Gerber H, Studer H. Naturally occurring clones of cells with high intrinsic proliferation potential within the follicular epithelium of mouse thyroids. Cancer Res 1987;47:1646–51. 3815361

[41] Fagman H , Andersson L, Nilsson M. The developing mouse thyroid: embryonic vessel contacts and parenchymal growth pattern during specification, budding, migration, and lobulation. Dev Dyn 2006;235:444–55. 16331648 10.1002/dvdy.20653

[42] Liang S , Johansson E, Barila G, Altschuler DL, Fagman H, Nilsson M. A branching morphogenesis program governs embryonic growth of the thyroid gland. Development 2018;145:dev146829. 29361553 10.1242/dev.146829 PMC5825846

[43] Nilsson M , Ericson LE. Effects of epidermal growth factor and phorbol ester on thyroid epithelial integrity. Exp Cell Res 1995;219:626–39. 7641814 10.1006/excr.1995.1273

[44] Baptist M , Dumont JE, Roger PP. Intercellular heterogeneity of early mitogenic events: cAMP generalizes the EGF effect on c-Fos protein appearance but not on MAP kinase phosphorylation and nuclear translocation in dog thyroid epithelial cells. Exp Cell Res 1995;221:160–71. 7589241 10.1006/excr.1995.1363

[45] Roger PP , Baptist M, Dumont JE. A mechanism generating heterogeneity in thyroid epithelial cells: suppression of the thyrotropin/cAMP-dependent mitogenic pathway after cell division induced by cAMP-independent factors. J Cell Biol 1992;117:383–93. 1313816 10.1083/jcb.117.2.383 PMC2289413

[46] Coclet J , Foureau F, Ketelbant P, Galand P, Dumont JE. Cell population kinetics in dog and human adult thyroid. Clin Endocrinol (Oxf) 1989;31:655–65. 2627756 10.1111/j.1365-2265.1989.tb01290.x

[47] Saad AG , Kumar S, Ron E, Lubin JH, Stanek J, Bove KE, . Proliferative activity of human thyroid cells in various age groups and its correlation with the risk of thyroid cancer after radiation exposure. J Clin Endocrinol Metab 2006;91:2672–7. 16670159 10.1210/jc.2006-0417

[48] Fiore APZP , Fuziwara CS, Kimura ET. High iodine concentration attenuates RET/PTC3 oncogene activation in thyroid follicular cells. Thyroid 2009;19:1249–56. 19725779 10.1089/thy.2008.0408

[49] Fuziwara CS , Kimura ET. High iodine blocks a Notch/miR-19 loop activated by the BRAF(V600E) oncoprotein and restores the response to TGFβ in thyroid follicular cells. Thyroid 2014;24:453–62. 23998804 10.1089/thy.2013.0398 PMC3949441

[50] Johansson E , Liang S, Moccia C, Carlsson T, Andersson D, Fagman H, . Asynchrony of apical polarization, luminogenesis, and functional differentiation in the developing thyroid gland. Front Endocrinol (Lausanne) 2021;12:760541. 34975747 10.3389/fendo.2021.760541 PMC8719337

[51] Westermark K , Nilsson M, Ebendal T, Westermark B. Thyrocyte migration and histiotypic follicle regeneration are promoted by epidermal growth factor in primary culture of thyroid follicles in collagen gel. Endocrinology 1991;129:2180–6. 1915099 10.1210/endo-129-4-2180

[52] Antonica F , Kasprzyk DF, Opitz R, Iacovino M, Liao X-H, Dumitrescu AM, . Generation of functional thyroid from embryonic stem cells. Nature 2012;491:66–71. 23051751 10.1038/nature11525 PMC3687105

[53] Studer H , Gerber H, Zbaeren J, Peter HJ. Histomorphological and immunohistochemical evidence that human nodular goiters grow by episodic replication of multiple clusters of thyroid follicular cells. J Clin Endocrinol Metab 1992;75:1151–8. 1400886 10.1210/jcem.75.4.1400886

[54] Nilsson M , Dahlman T, Westermark B, Westermark K. Transforming growth factor-beta promotes epidermal growth factor-induced thyroid cell migration and follicle neoformation in collagen gel separable from cell proliferation. Exp Cell Res 1995;220:257–65. 7556432 10.1006/excr.1995.1314

[55] Postiglione MP , Parlato R, Rodriguez-Mallon A, Rosica A, Mithbaokar P, Maresca M, . Role of the thyroid-stimulating hormone receptor signaling in development and differentiation of the thyroid gland. Proc Natl Acad Sci U S A 2002;99:15462–7. 12432093 10.1073/pnas.242328999 PMC137739

[56] Dame K , Cincotta S, Lang AH, Sanghrajka RM, Zhang L, Choi J, . Thyroid progenitors are robustly derived from embryonic stem cells through transient, developmental stage-specific overexpression of Nkx2-1. Stem Cell Rep 2017;8:216–25. 10.1016/j.stemcr.2016.12.024 PMC5312259 28162994

[57] Nilsson M , Fagman H. Development of the thyroid gland. Development 2017;144:2123–40. 28634271 10.1242/dev.145615

[58] Kluge B , Renault N, Rohr KB. Anatomical and molecular reinvestigation of lamprey endostyle development provides new insight into thyroid gland evolution. Dev Genes Evol 2005;215:32–40. 15592682 10.1007/s00427-004-0450-0

[59] Yamagishi M , Huang T, Hozumi A, Onuma TA, Sasakura Y, Ogasawara M. Differentiation of endostyle cells by Nkx2-1 and FoxE in the ascidian Ciona intestinalis type A: insights into shared gene regulation in glandular- and thyroid-equivalent elements of the chordate endostyle. Cell Tissue Res 2022;390:189–205. 36048302 10.1007/s00441-022-03679-w

[60] Takagi W , Sugahara F, Higuchi S, Kusakabe R, Pascual-Anaya J, Sato I, . Thyroid and endostyle development in cyclostomes provides new insights into the evolutionary history of vertebrates. BMC Biol 2022;20:76. 35361194 10.1186/s12915-022-01282-7 PMC8973611

[61] Boucai L , Seshan V, Williams M, Knauf JA, Saqcena M, Ghossein RA, . Characterization of subtypes of BRAF-mutant papillary thyroid cancer defined by their thyroid differentiation score. J Clin Endocrinol Metab 2022;107:1030–9. 34897468 10.1210/clinem/dgab851 PMC8947218

[62] Cancer Genome Atlas Research Network . Integrated genomic characterization of papillary thyroid carcinoma. Cell 2014;159:676–90. 25417114 10.1016/j.cell.2014.09.050 PMC4243044

[63] An JH , Song K-H, Kim SK, Park KS, Yoo YB, Yang J-H, . RAS mutations in indeterminate thyroid nodules are predictive of the follicular variant of papillary thyroid carcinoma. Clin Endocrinol (Oxf) 2015;82:760–6. 25109485 10.1111/cen.12579

[64] Hymowitz SG , Malek S. Targeting the MAPK pathway in RAS mutant cancers. Cold Spring Harb Perspect Med 2018;8:a031492. 29440321 10.1101/cshperspect.a031492 PMC6211377

[65] Moore AR , Rosenberg SC, McCormick F, Malek S. RAS-targeted therapies: is the undruggable drugged? Nat Rev Drug Discov 2020;19:533–52. 32528145 10.1038/s41573-020-0068-6 PMC7809886

[66] Boucai L , Saqcena M, Kuo F, Grewal RK, Socci N, Knauf JA, . Genomic and transcriptomic characteristics of metastatic thyroid cancers with exceptional responses to radioactive iodine therapy. Clin Cancer Res 2023;29:1620–30. 36780190 10.1158/1078-0432.CCR-22-2882 PMC10106408

[67] Aeschimann S , Kopp PA, Kimura ET, Zbaeren J, Tobler A, Fey MF, . Morphological and functional polymorphism within clonal thyroid nodules. J Clin Endocrinol Metab 1993;77:846–51. 8370709 10.1210/jcem.77.3.8370709

[68] Nelson CM . On buckling morphogenesis. J Biomech Eng 2016;138:021005. 26632268 10.1115/1.4032128 PMC4844087

