# Supplementary material for: Tumor Cell Plasticity and Stromal Microenvironment Distinguish Papillary and Follicular Growth Patterns in a Mouse Model of BRAFV600E-Induced Thyroid Cancer
Source: Cancer Res Commun. 2025 Mar 7;5(3):409–21. doi: 10.1158/2767-9764.CRC-24-0474 (PMC11885905; doi:10.1158/2767-9764.CRC-24-0474)
Supplement: Figure S1 — IF images Figure S1. Clonal tracing of sporadically developed BRAF mutant neoplasia in mouse thyroid. [file crc-24-0474_figure_s1_suppsf1.pdf]

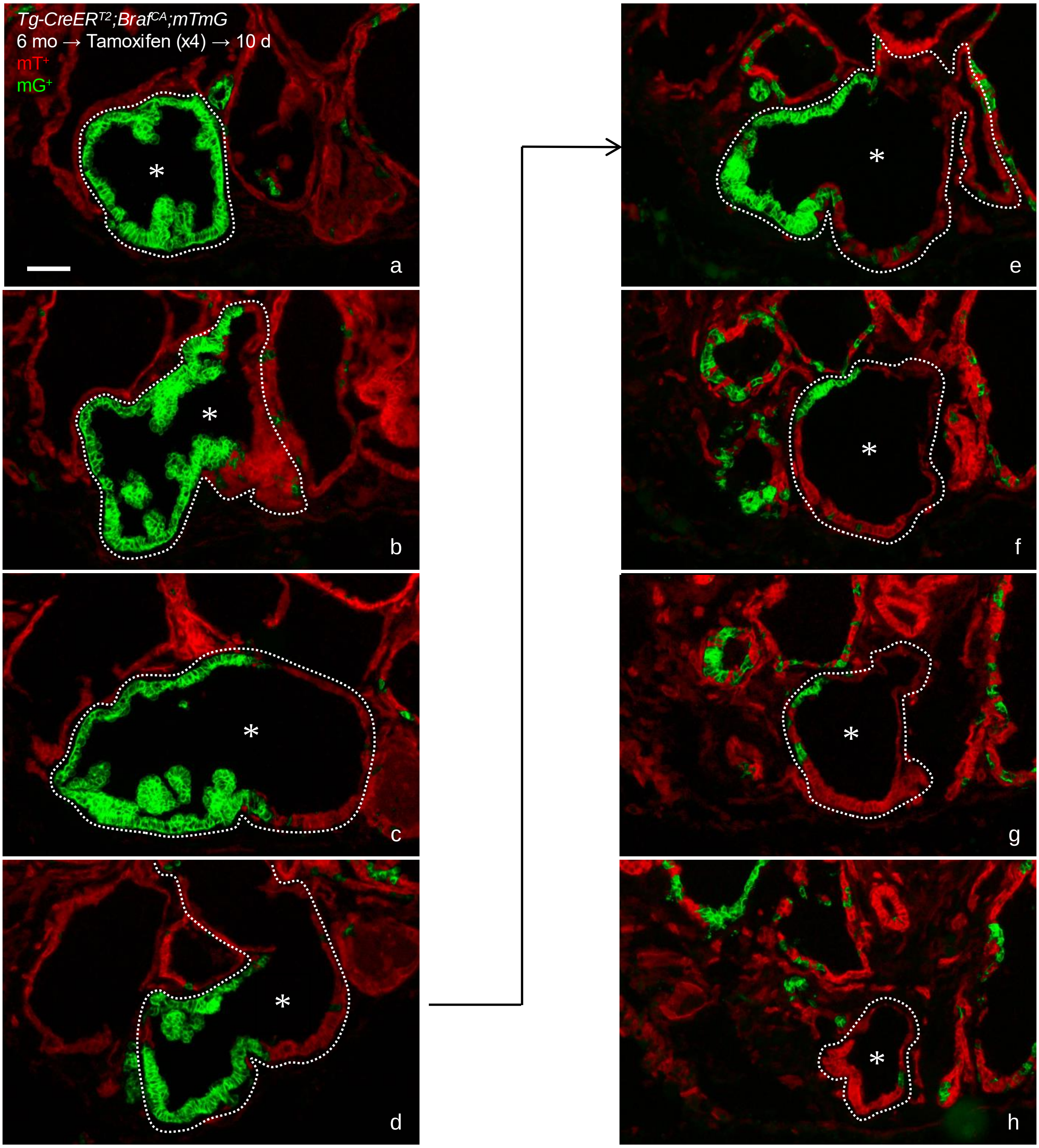

Supplementary Fig. S1. Clonal tracing of sporadically developed BRAF mutant neoplasia in mouse thyroid. Tracing of BRAF mutant clones by reporter gene activation in 6 months (mo) old *Tg-CreER^{T2};Braf^{CA};mTmG* mice. Tamoxifen (Tam) was injected once daily x4 starting 10 days (d) before sacrifice; only non-mutant thyroid cells expressing CRE are assumed to respond to induction.

a-h Thyroid tissue images from consective serial sections (of which c is identical to Fig. 2g). The encircled follicle is neoplastic and consists of mG$^+$ and mT$^+$ mutant clones of which the former is in all probability lineage-traced due to spontaneous *mTmG* and *Braf^{CA}* co-activation taking place long before the induced activation. Asterisks indicate irregular lumen of the follicle. Whereas the encircled mT$^+$ clone is non-responsive, most other follicles display variable number of mG$^+$ cells. See Fig. 2 and associated text in Results for further explanations and comments. Bar: 50 µm (applies to all images).
