# Supplementary material for: Tumor Cell Plasticity and Stromal Microenvironment Distinguish Papillary and Follicular Growth Patterns in a Mouse Model of BRAFV600E-Induced Thyroid Cancer
Source: Cancer Res Commun. 2025 Mar 7;5(3):409–21. doi: 10.1158/2767-9764.CRC-24-0474 (PMC11885905; doi:10.1158/2767-9764.CRC-24-0474)
Supplement: Figure S2 — IF images Figure S2. Clonal tracing of BRAF mutant thyroid carcinoma that displays a follicular tumor phenotype. [file crc-24-0474_figure_s2_suppsf2.pdf]

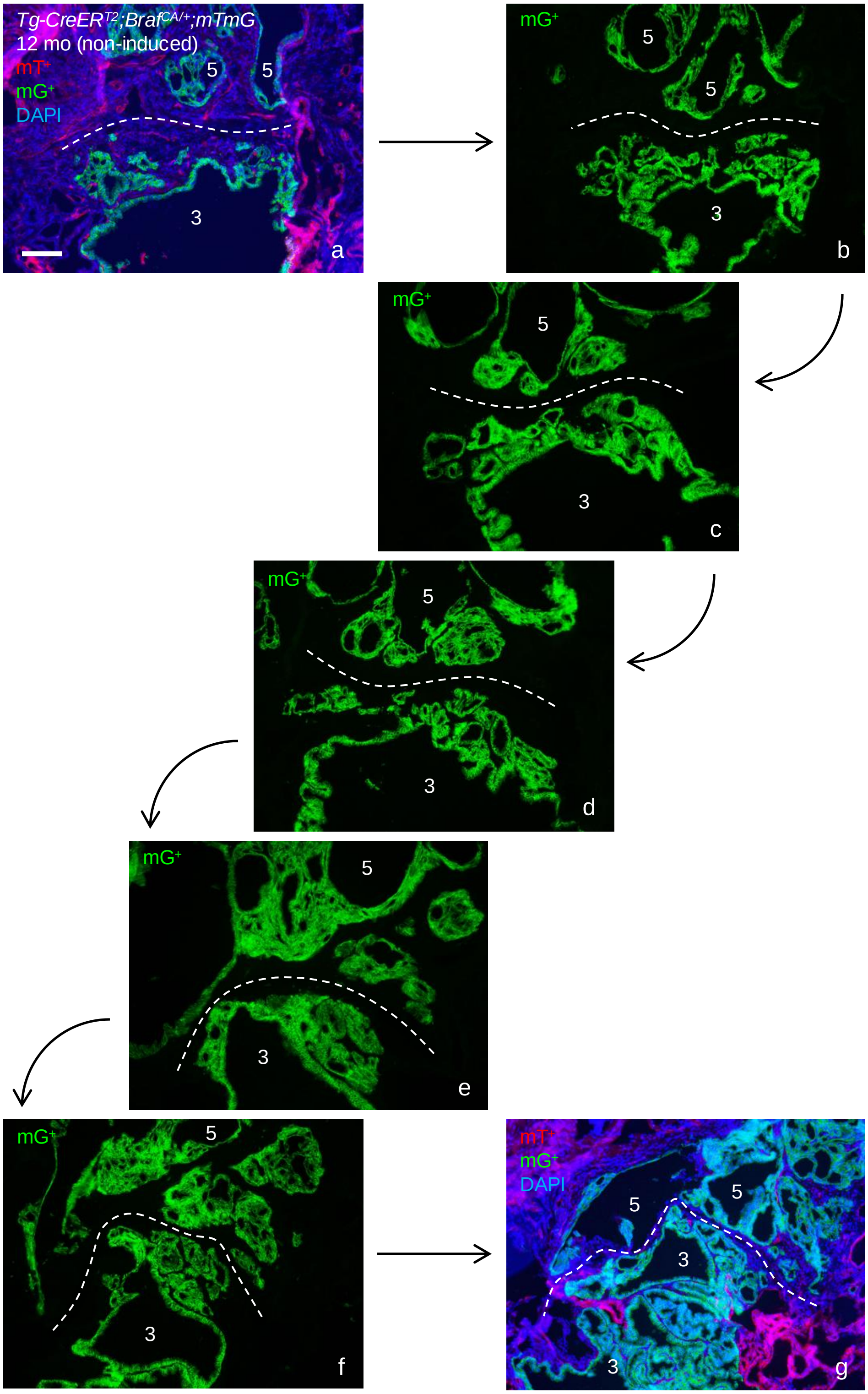

Supplementary Fig. S2. Clonal tracing of BRAF mutant thyroid carcinoma that displays a follicular tumor phenotype. Retrieved from serial sections of a 12 months (mo) old *Tg-CreER*^{T2}*;Braf*^{CA}*;mTmG* mouse subjected to spontaneous *Braf*^{CA} and reporter gene co-activation (identical to the animal and thyroid sample shown in Fig. 5). **a-g** Image stack of adjacent tumor clones 3 and 5 (of which c is identical to Fig. 5c´); images in b-f show green channel only for improved visibility of the gap consisting of stromal tissue (outlined) that separates the two clones. See Fig. 5 and associated text in Results for further details and comments. Bar: 100 µm (applies to all images).
